# Direct assessment of mental health and metabolic syndrome amongst Indonesian adolescents: a study design for a mixed-methods study sampled from school and community settings

**DOI:** 10.1080/16549716.2020.1732665

**Published:** 2020-03-16

**Authors:** Peter S. Azzopardi, Lisa Willenberg, Nisaa Wulan, Yoga Devaera, Bernie Medise, Aida Riyanti, Ansariadi Ansariadi, Susan Sawyer, Tjhin Wiguna, Fransiska Kaligis, Jane Fisher, Thach Tran, Paul A. Agius, Rohan Borschmann, Alex Brown, Karly Cini, Susan Clifford, Elissa C. Kennedy, Alisa Pedrana, Minh D. Pham, Melissa Wake, Paul Zimmet, Kelly Durrant, Budi Wiweko, Stanley Luchters

**Affiliations:** a Global Adolescent Health Group, Maternal, Child and Adolescent Health Program, Burnet Institute, Melbourne, Australia; b Department of Paediatrics, Royal Children's Hospital, The University of Melbourne, Melbourne, Australia; c Aboriginal Health Equity Theme, South Australian Health and Medical Research Institute, Adelaide, Australia; d Population Health Group, Murdoch Children’s Research Institute, Melbourne, Australia; e Department of Child Health, Universitas Indonesia, Jakarta, Indonesia; f Department of Obstetrics and Gynaecology, Faculty of Medicine, Universitas Indonesia, Jakarta, Indonesia; g Department of Epidemiology, School of Public Health, Universitas Hasanuddin, Makassar, Indonesia; h Department of Psychiatry, Faculty of Medicine, Universitas Indonesia, Jakarta, Indonesia; i Global and Women’s Health Unit, School of Population and Preventive Medicine, Monash University, Melbourne, Australia; j Maternal, Child and Adolescent Health Program, Burnet Institute, Melbourne, Australia; k Justice Health Unit, Centre for Health Equity, Melbourne School of Population and Global Health, University of Melbourne, Melbourne, Australia; l Disease Elimination Program, Burnet Institute, Melbourne, Australia; m Department of Diabetes, Central Clinical School, Monash University, Melbourne, Australia; n Research and Social Services, Faculty of Medicine, Universitas Indonesia, Jakarta, Indonesia

**Keywords:** Study design, objective assessment, mental disorder, metabolic syndrome, adolescents, school-based, community-based, Indonesia

## Abstract

Non-communicable diseases (NCDs) are the leading cause of morbidity and mortality globally, with the burden largely borne by people living in low- and middle-income countries. Adolescents are central to NCD control through the potential to modify risks and alter the trajectory of these diseases across the life-course. However, an absence of epidemiological data has contributed to the relative exclusion of adolescents from policies and responses. This paper documents the design of a study to measure the burden of metabolic syndrome (a key risk for NCDs) and poor mental health (a key outcome) amongst Indonesian adolescents. Using a mixed-method design, we sampled 16–18-year-old adolescents from schools and community-based settings across Jakarta and South Sulawesi. Initial formative qualitative enquiry used focus group discussions to understand how young people conceptualise mental health and body weight (separately); what they perceive as determinants of these NCDs; and what responses to these NCDs should involve. These findings informed the design of a quantitative survey that adolescents self-completed electronically. Mental health was measured using the Centre for Epidemiologic Studies Depression Scale-Revised (CESD-R) and Kessler-10 (both validated against formal psychiatric interview in a subsample), with the metabolic syndrome measured using biomarkers and anthropometry. The survey also included scales relating to victimisation, connectedness, self-efficacy, body image and quality of life. Adolescents were sampled from schools using a multistage cluster design, and from the community using respondent-driven sampling (RDS). This study will substantially advance the field of NCD measurement amongst adolescents, especially in settings like Indonesia. It demonstrates that high quality, objective measurement is acceptable and feasible, including the collection of biomarkers in a school-based setting. It demonstrates how comparable data can be collected across both in-school and out of school adolescents, allowing a more comprehensive measure of NCD burden, risk and correlates.

## Background

### The need for high-quality direct assessment of NCD in adolescents

Non-communicable diseases (NCDs) are now the leading causes of death and disability globally [1]. People living in low- and middle-income countries (LMICs) are disproportionately affected, with NCDs in these settings contributing a large financial burden through lost productivity and healthcare, posing a substantial barrier to sustainable development [2]. While adults carry the burden of NCD deaths and disability, adolescents are central to NCD control and represent an important target for intervention [3]. Firstly, key risks for NCDs (including substance use, unhealthy diet, physical inactivity, high body mass and components of the metabolic syndrome) typically rise during adolescence and are potentially modifiable during this developmental stage [4]. Secondly, key NCD outcomes including mental disorders (particularly depression and anxiety) and substance use disorders (both licit and illicit) emerge during adolescence [5]. Untreated, these NCDs can disrupt education, increase the risk of incarceration and/or result in social isolation, compounding the socio-economic disadvantage that often co-exists with NCDs [6]. Thirdly, for those who become parents, NCDs during adolescence increase the risk of NCDs in offspring and, as such, adolescence provides a unique window to disrupt the intergenerational cycles of transmission [7].

Yet adolescents have remained very much at the margins of NCD policy, programming and investment [8]. One reason may be the paucity of data measuring the burden of NCDs in adolescents, resulting in these needs remaining invisible to those responsible for policy, programming and investments. For example, the global coverage of minimally sufficient data for mental disorders amongst children and adolescents is only 6.5%; 124 countries, including populous countries like Indonesia, have no usable data [9]. A major challenge to collecting good quality data around NCDs in adolescents is the availability of high quality, validated scales that are sensitive to local culture and language. Other important contributors to NCDs in adolescence, such as metabolic syndrome, are typically asymptomatic and require direct assessment; the collection of blood is a common barrier to these assessments in population-based samples of adolescents [10]. Further complicating the scenario, adolescents who are likely most at risk of NCDs (such as those who are homeless, incarcerated or disengaged from school) are typically not reached through household or school-based surveys, the typical sampling frames for adolescent health surveys [11].

Understanding opportunities for NCD prevention is a recognised policy priority of Indonesia [12], a country where rapid socioeconomic development and urbanisation has driven a substantial epidemiological transition [13]. While the burden of NCDs amongst Indonesian adolescents remains poorly measured, data from other rapidly urbanising populations suggest the burden is likely to be substantial [14]. Given its large population (268 million people, 65 million adolescents), understanding and addressing the burden of NCDs in Indonesian adolescents is of global significance [5]. This study design paper documents a mixed-methods research study design with the aim of collecting high-quality data on the key NCD outcomes and risks amongst adolescents in Indonesia. Funded by the Australia–Indonesia Centre (a partnership between the Australian and Indonesian governments), it was undertaken to gain a better understanding of the opportunities for primary prevention of NCDs early in the life-course.

### Collaborative co-design of the research study and rationale for study focus

Six investigators (PA and SL from Australia and BW, YD, BM and AR from Indonesia) participated in a 2-day meeting in Jakarta in January 2017 that underpinned the co-design of the research project. The decision was made to focus the study on mental health given mental disorder contributes almost a quarter of the modelled disease burden amongst Indonesian adolescents [15]. Mental health was also identified as an area of great policy relevance but where primary data were very limited [16]. Metabolic syndrome was additionally selected as a focus given the rapidly increasing prevalence of obesity in Indonesia [5], and unpublished data showing a higher than expected prevalence of impaired glucose tolerance amongst Indonesian secondary school students (Personal communication, Wiweko 2017). There is also emerging evidence suggesting mental disorder and metabolic syndrome are inter-related, potentially sharing common determinants of stress and/or circadian rhythm disruption [17,18]. We noted that while the prevalence of tobacco smoking amongst Indonesian adolescents is amongst the highest globally [5], there are good quality primary data for this specific NCD risk in Indonesia [19]. As such, we measured tobacco smoking (as well as alcohol and illicit substance use) but did not have these as the primary focus of this research.

We focussed this study on high-quality quantitative assessment of mental health and metabolic syndrome. To enable this, we included a formative qualitative phase to understand the context and perspectives of Indonesian adolescents so as to inform targets for quantitative assessment, as well as targets for effective policy and programmatic responses. For example, the Wellbeing of Adolescents in Vulnerable Environments (WAVE study, adolescent health in mega-cities – but not including Indonesia) focussed measures of mental health around hope for the future and depression, suicidal ideation and post-traumatic stress disorder (PTSD) [20]. The investigator team was unsure how these concepts aligned with the mental health needs of Indonesian adolescents, particularly PTSD.

Obtaining a representative sample of adolescents across Indonesia (34 provinces across many islands) was beyond the scope of the existing project. We therefore sampled adolescents from urban, peri-urban and remote areas of Indonesia in order to gain some understanding of variation by geography and socioeconomic development. We also specifically sampled adolescents not engaged in school to explore of these adolescents were at greater risk of NCDs.

### Aims

The overarching aim of this study was to measure the prevalence of common mental disorders (a key NCD outcome) and the metabolic syndrome (a key risk) amongst Indonesian adolescents with the goal of informing future NCD policy and programming. Specifically, we aimed to:

1. Qualitatively explore how in and out of school Indonesian adolescents conceptualise mental disorders and metabolic risk (separately), their perceptions of the determinants, and their recommendations around appropriate responses;
2. Quantitatively measure the population prevalence of mental disorder and metabolic syndrome, their correlates and inter-relationship amongst in- and out-of-school Indonesian adolescents.

### Methods

The study was mixed-methods in design and sequential. We first undertook qualitative enquiry using focus group discussions with in- and out-of-school adolescents, exploring mental disorder and metabolic risk separately. We then undertook a representative quantitative survey, with biomarker assessment and psychiatric interview in some adolescents, to estimate the prevalence of mental disorder and metabolic risk amongst in- and out-of-school adolescents aged 16–18 years in Indonesia (Table 1).

**Table 1.** Summary of study design

	Qualitative		Quantitative				
	Body weight FGDs	Mental health FGDs	Self-report survey	Anthropometry height, weight, waist circumference	Biomarkers Serum samples, blood pressure	Psychiatric interview (MINI Kids)	Online diet diary KUALA24
**Jakarta**							
In-school	✔	✔	✔	✔	✔	✔ (subset)	✔
Out-of-school	✔	✔	✔	✔			
**South Sulawesi**							
In-school	✔	✔	✔	✔			
Out-of-school	✔	✔	✔	✔			

## Study sites and participants

The study was conducted in Indonesia, a majority Muslim country. Two provinces were purposively selected to capture Indonesia’s geographic and socio-economic diversity. Jakarta, the capital city with a population of just above 10 million, was selected as it represents the most developed and populous province. South Sulawesi (Gowa Regency, situated in the mountainous region on the south-western peninsular of Sulawesi Island) was selected to sample adolescents living in peri-urban and more remote regions of Indonesia. Jakarta and South Sulawesi differ substantially by population density (15366/km^2^ vs 397/km^2^); population size of 15–19 year olds (706,550 vs 68,112); and Human Development Index (80.06 vs 68.33) [21,22].

Given the focus on mental disorder and metabolic syndrome, the study was focussed on adolescents aged greater than 16 years given this marks an important transition in mental health and metabolic risk, capacity to provide consent, and capacity to explore complex issues in research [3,23]. We set the upper age threshold at 18 years given this is typically when adolescents complete secondary education in Indonesia and transition out of education (an important sampling frame of this study because it is also an important platform for health intervention) [3]. The dynamic nature of the burden of NCDs across adolescence also influenced our decision on a narrow age-band for this study [3].

## Community engagement

We invested in extensive community engagement given the sensitive nature of the study’s focus, but also to ensure that the study was appropriate and relevant to local context. Community forums (attended by youth advocacy groups, parents, school staff, community health service staff and community leaders) were held separately in Jakarta and South Sulawesi in early 2017 (during study development) and again in 2018 (prior to embarking on quantitative data collection) to discuss the study, its aims and procedures. Additional meetings were held with school principals in Jakarta given the inclusion of blood sampling and psychiatric interview in these settings, as detailed below.

## Study governance and communication

This research study was a cross-country collaboration between researchers and institutions in Indonesia and Australia. The study was co-led by principal investigators from Australia (PA and SL) and Indonesia (BW), with the investigator team consisting of researchers and clinicians from Jakarta, Makassar and Melbourne. Fortnightly investigator meetings were held by videoconference, these meetings occurred weekly during data collection. The research team also developed a WhatsApp group to facilitate communication, particularly during data collection and to co-ordinate activities. Two distinct teams of data collectors were assembled to enable parallel data collection across the two jurisdictions, with senior research assistants and investigators involved across both groups to ensure consistency in method. The research team in South Sulawesi developed a ‘team uniform’ that was worn during data collection, additionally helping to build team camaraderie, but also helping to identify researchers within the rural school and community settings.

## Design: qualitative enquiry

The formative qualitative phase used focus group discussions (FGDs) so as to capture a broad range of perspectives and stimulate discussion amongst adolescents. Given that metabolic syndrome is a cluster of symptoms, many of which are inconspicuous, we focussed these discussions on the concept of body weight, specifically being overweight to ensure consistency and a shared meaning for participants.

### Interview guide

A semi-structured question guide was used to facilitate each FGD, based on consultation with local partners and review of similar studies. Each FGD began with open-ended questions to explore how adolescents conceptualised mental health or body weight (Table 2). Participants were instructed not to talk about their own experiences but to discuss the issue more broadly. A diagram of a socio-ecological framework (levels included individual, friends and peers, family, school, social media, and community) was then used to guide participants’ discussions about perceived determinants. Participants finally discussed current approaches to mental health/body weight and proposed what an ideal response would include.

**Table 2.** Question guide for the conceptualisation of mental health/body weight

Mental health		Body weight	
Question	*Probes*	Question	Probes
What do you think of when you hear the word ‘mental health’?	What do you think it means to be mentally well/have good mental health? Why do you think being mentally well is important?	What is your understanding of ‘healthy’ or ‘normal’ weight?	How do you know if a person is healthy? What do you consider a healthy weight?
What types of behaviours and emotional states do you associate with people who have good mental health?	How do people who are mentally well behave? What do people who are mentally well look like?		
What do you think of when you hear the term ‘poor mental health/mentally unwell’?	How would you describe poor mental health? What terms have you heard other people use to describe poor mental health?	What is your understanding of ‘above normal’ weight’?	How do you know if a person is above normal weight? When do you think a person is considered above normal weight/unhealthy weight?
What types of behaviours and emotional states do you associate with people who have poor mental health/are mentally unwell?	How do people with poor mental health behave? What do people who are mentally unwell look like? How would you know if someone you knew had poor mental health?	What types of physical features do you associate with above normal body weight? What types of behaviours do you associate with someone who is above normal body weight?	
Do you think poor mental health is an issue for adolescents in Indonesia? Why/why not?	In what ways do you think poor mental health impacts physical health? In what ways do you think poor mental health impacts on relationships between people – e.g. family, friends/peers, colleagues? In what ways do you think poor mental health would impact schooling/education, free time employment?	Do you think being above normal weight is an issue for adolescents in Indonesia? Why/why not?	In what ways do you think above normal weight impacts general health? Psychological health? In what ways do you think above normal weight impacts on relationships between people – e.g. family, friends/peers, colleagues? In what ways do you think above normal weight would impact schooling/education, employment?
What kinds of attitudes/behaviours do you think people have towards individuals with poor mental health? What do you think influences these attitudes/behaviours?	Are there any cultural or religious beliefs that influence these attitudes/behaviours? Are people with poor mental health accepted by the community? Do they face any stigma or discrimination? How do you think this stigma/discrimination impacts on them?	What kinds of attitudes/behaviours do you think people have towards someone who is above normal body weight? What do you think influences these attitudes/behaviours?	Are there any cultural or religious beliefs that influence these attitudes/behaviours? Are people who are above normal body weight accepted by the community? Do they face any stigma or discrimination? How do you think this stigma/discrimination impacts on them?

The question guide was developed in English, translated into Bahasa Indonesia and then back-translated to check the accuracy of the translation. The guides were piloted with a mixed group of school and community-based adolescents (8 in total). The main modifications related to the mental health FGDs. There was some confusion over the term ‘wellbeing’, as no direct translation exists in Bahasa Indonesia. We subsequently modified the question guides to refer to ‘good mental health’ and ‘poor mental health’. The first two FGDs were also observed by study investigators (LW, BM, YD and AR). We found that the conceptualisation of mental health largely focussed on poor mental health rather than good mental health. For consistency across FGDs, we focussed the discussion of determinants around ‘poor mental health’, and then specifically on stress and depression as these constructs were consistent with the symptoms and issues discussed. Social media also emerged as an important determinant, and this was subsequently added to the socio-ecological framework.

### Sampling strategy and recruitment

For the school sample in Jakarta, participants were purposively selected from a public, a private and a religious school that the research team had established relationships with. Each school principal identified eligible students (equal numbers of males and females) who may have a range of perspectives to share. Eligible students were 16–18 years old, enrolled in grades 10–12, and had attended school in the preceding 90 days. Students from across schools were then invited to participate in FGDs held at the Indonesian Medical Education and Research Institute (IMERI), a central and easily accessible location in Jakarta, with separate FGDs for males and females (to observe cultural sensitivity and encourage open discussion). In South Sulawesi, students were recruited using the same eligibility criteria from a single state school with FGDs held at that school.

Community-based participants in Jakarta were purposively selected from internet cafes and social institutions (government-funded training centres for out-of-school adolescents, including those experiencing homelessness). Eligible participants were 16–18 years old and were either not enrolled in school or had not attended school in the preceding 90 days. Permission was sought from the manager of each of these settings, after which the research team visited each one to identify eligible young people who were then invited to participate. FGDs were held at IMERI. In South Sulawesi, eligible participants were identified through a community health worker as internet cafes and social institutions were fewer. Interested potential participants were provided with a leaflet that outlined the details of the FGD and were provided with 1 week to obtain parent or guardian consent. These FGDs were held at a school during holiday break.

Written informed consent of FGD participants was obtained from respective parents or guardians, with adolescents themselves also providing written informed consent/assent. Further, all participants provided verbal consent at the beginning of each audio-recorded FGD.

### Procedure

FGDs were conducted in September 2017. Sixteen FGDs were undertaken to accommodate the two study locations (Jakarta and South Sulawesi), the two distinct topics (mental health and metabolic syndrome), males and females separately, and the two settings (school and community). Preliminary analysis indicated that data saturation had been reached so no further FGDs were conducted. A minimum of 8 and a maximum of 12 adolescents were invited to participate in each FGD to maximise opportunities for sharing opinions within the group. They were facilitated by two Indonesian researchers who were of the same gender and from the same province as the participants; these researchers had experience in qualitative research and received further training from the investigator team around the study’s aims, qualitative methods, participatory research with adolescents, and research ethics. FGDs were conducted in Bahasa Indonesia in Jakarta and in local dialect in South Sulawesi. Each FGD began with an ‘ice-breaker’ to facilitate open discussion and lasted 45–60 min. In addition to interviews being digitally audio-recorded, one facilitator took written notes throughout each FGD. Participants were provided with a light refreshment and reimbursed for travel costs if required.

### Analysis

Audio recordings were transcribed in Bahasa Indonesia and then translated into English. Ten per cent were back-translated to check for accuracy. Transcripts were thematically analysed by two researchers using an inductive approach. Transcripts were read and re-read to inform the initial coding frame. Researchers then individually coded transcripts and met regularly to review the coding frame, refining and adding new codes as needed to form the final coding framework. Transcripts were then coded using NVivo11 software and a summary of the data entered into the framework. Original audio recordings were reviewed where clarification was required. The framework was then reviewed to identify key themes and sub-themes and relationships between these. Quotes were recorded to illustrate key themes. Findings were validated with the field research teams and with in-country partners.

## Design: quantitative assessment

The quantitative assessment involved a representative cross-sectional survey of adolescents aged 16–18 years both in- and out-of-school populations. Data were collected using an electronic tablet-administered self-report instrument that all participants were invited to undertake. All participants were invited to have basic anthropometric measurements taken, with adolescents sampled from schools in Jakarta additionally invited to have biomarkers collected, have a formal psychiatric interview, and complete an online nutrition diary (Table 1).

### Measures

#### Self-administered survey

The investigator team considered several approaches to data collection for the survey. Firstly, we considered how to administer the survey. There was some concern that adolescents would not complete a survey that was self-administered, either because the in-school adolescents would not take it seriously during class, or due to concerns of limited literacy for the community-based participants. We considered using trained interviewers rather than a self-completed survey, which was weighed against the cost (personnel, time) and the potential response-bias that this would introduce which was thought to be especially relevant given the sensitive nature of mental health in Indonesia. On balance, we decided that the survey would be self-administered, with trained interviewers available to assist where required (for example, limited literacy). We next considered the mode of the survey. While paper-based surveys are commonly used in Indonesia (including the Global School Health Survey), we decided to use electronic data capture with the belief that this would be more engaging and efficient. Electronic capture also eliminated potential data entry error and enabled real-time storage and analysis of data across Australia and Indonesia through a secure cloud. Advan i7d tablets running the 6.0 Marshmallow operating system were used, with the survey developed using RedCAP. The survey was developed by the research team, with the requirement that it could be administered in a single class (less than 60 min). The draft survey was then extensively tested by the research team, as well as by an Indonesian research student who was independent of the study, which resulted in trimming of content. The survey was further refined following piloting with the young Indonesian research assistants who were employed to facilitate data collection.

Emerging themes from the mental health FGDs identified that adolescents commonly viewed mental health needs within the constructs of ‘stress’ and depression which informed the decision to include the Kessler Psychological Distress Scale (K10, a measure of psychological distress) and the Centre for Epidemiological Studies Depression Scale – Revised (CESD-R) scale [24,25]. The additional value of the K10 scale is that it is widely used globally, while the CESD-R was also used in the WAVE study of adolescents in mega-cities, which facilitates comparison [20]. In response to adolescents’ other perspectives of determinants of mental health that were apparent from the qualitative analyses, we also included scales of victimisation, connectedness, and self-efficacy (Table 3). These analyses also highlighted the interconnectedness between community safety, injuries and mental health, which resulted in us including appropriate scales to measure these constructs. Qualitative analyses of body weight from the FGDs informed the inclusion of measures of body image and quality of life, including physical function.

**Table 3.** Overview of quantitative measures included in the self-report survey

Theme	Tool name or source	Domain	Items	Description
**Mental health**	Kessler 10 (K10) [25]	Psychological distress	10	Widely used measure of psychological distress amongst adolescents, assessing symptoms over the past 4 weeks. Responses to each item are on a 5-point Likert scale, summed to provide a summary score.
	Centre for Epidemiological Studies Depression Scale Revised (CESD-R) [24]	Depression	20	Screening tool for symptoms of depression (last 2 weeks) aligned with the Diagnostic and Statistical Manual V.
**Intentional and unintentional injury**	Sourced from Global School Health Survey [34]	Physical injuries	4	Questions relating to injuries sustained in the last 12 months, including major cause, help-seeking behaviour and the influence of substance use.
	Sourced from Youth Risk Behaviour Survey [39]	Road traffic injuries and safety	4	Questions relating to motor vehicle and cycle injuries, including safety, influence of substance and mobile phone use.
		Self-harm	3	History of deliberate self-harm (ever, last 12 months) and frequency.
**Quality of life**	Youth Quality of Life Instrument-Surveillance Version (YQoL-S) [40]	Quality of life	13	Multidimensional tool that asses generic quality of life of adolescents aged 11 to 18 years.
	The Pediatric Quality of Life Inventory (PedsQL) [41]	Health-related quality of life (physical function subscale)	8	Physical function sub-scale, assessing physical ability and symptoms over preceding 30 days.
**Body perception**	The Body Dissatisfaction Scale [42]	Body dissatisfaction	3	Visual scales assessing ideal body type and actual body shape. The discrepancy between the actual versus ideal body shape constitutes the participant’s body dissatisfaction score.
**Nutritional risk**	Sourced from Health Behaviour in School-aged Children Survey (HBSC) [43,44]	Dietary intake	4	Questions relating to weekly consumption of fruits, vegetables, sweets and soft drinks.
		Physical activity	4	Questions relating to engagement in daily physical activity (over the last 7 days) and sedentary behaviours
**Substance abuse**	Sourced from Global Youth Tobacco Survey (GYTS) [45]	Tobacco use	9	Questions relating to experimentation, age at debut, current use, past use, cessation and advertising – including items around electronic cigarettes.
	Youth Risk Behaviour Surveillance System (YRBSS) [39]	Alcohol use	4	Questions relating to experimentation, age at debut, current use, alcohol-related issues.
		Illicit drug use	4	Questions relating to experimentation, age at debut, current use, drug type, drug-related problems.
**Victimisation**	The Juvenile Victimisation Questionnaire (JVQ) [46]	Polyvictimisation	12	Assessment of multiple forms of victimisation, including physical and emotional maltreatment, neglect, robbery, theft, vandalism, threat or assault, peer or sibling victimisation, family or community violence and exposure to gun shooting, bombing or cyber-bullying
**Self-efficacy**	Generalised Self-efficacy Scale [47]	Self-efficacy	10	Measure of perceived ability/belief in oneself to solve problems and reach goals.
**Connectedness**	Social Connectedness Scale (Revised) [48]	Social connectedness	6	Assesses the degree to which participants felt connected to others in their social environment
	Family Attachment Scale [49]	Family connectedness	4	Assesses connection to, and thoughts and feeling about, their mother and father.
**Safety**	Neighbourhood Scale [50]	Community safety	3	Respondents asked to rate levels of neighbourhood safety using a 5-level Likert scale.
**Health service access**	Adapted from Global School Health Survey [34] and Global Youth Tobacco Survey (GYTS) [45]	Barriers and enablers to health service access	20	Questions relating to health-seeking behaviours (physical & mental health), health information provision, preferred sources of information, health promotion messaging.

#### Anthropometry

All participants were weighed once (without shoes, dressed in light clothing) using Seca 877 digital scales, with measurements recorded to the nearest 100 g. Standing height was measured using a rigid stadiometer (Shorrboard portable). Two readings were taken, with a third taken if the two readings differed by more than 0.5 cm. Waist circumference was measured (light clothing, empty pockets) using a SECA 201 constant tension tape. Two readings were taken, with a third if the readings differed by more than 1.0 cm.

#### Biomarkers

**Figure 1.**
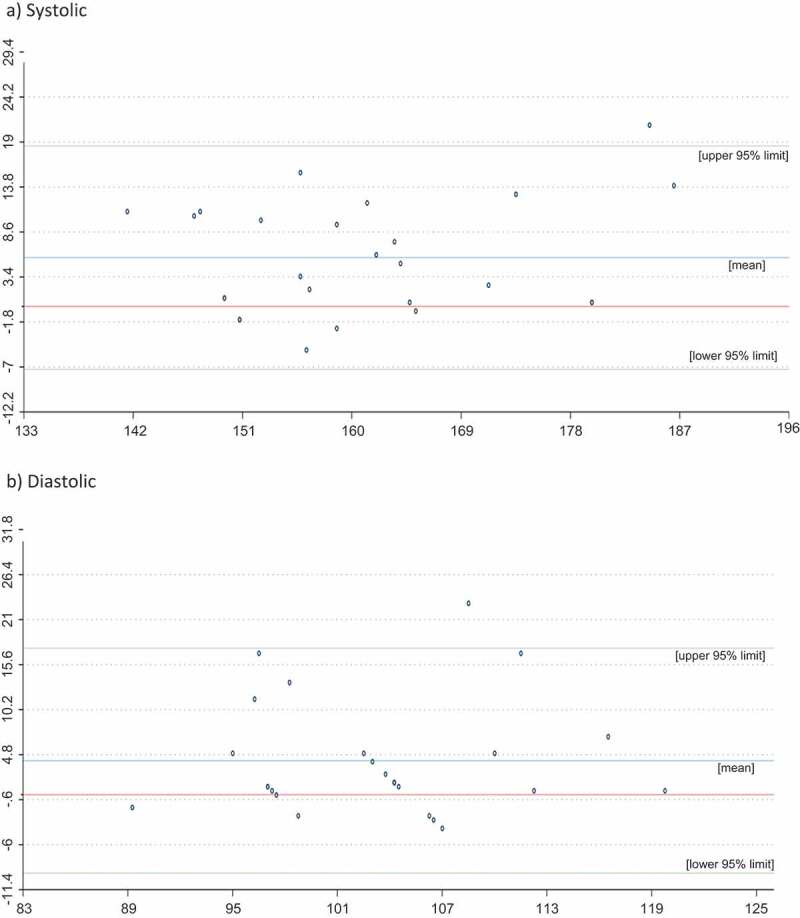
Bland-Altmann curves for systolic (upper panel) and diastolic blood pressure (lower panel) measured by physician (gold standard) and automated wrist sphygmomanometer in mmHg

Serum biomarkers included those required to measure the metabolic syndrome (Table 4), haemoglobin (as a screen for anaemia) and vitamin D. Biomarker assessment was only offered to school-based participants in Jakarta to ensure we could adequately follow up any abnormal result. Samples (non-fasting) were taken on the day of the main survey for pragmatic reasons, and as such, HbA1 c was measured rather than blood glucose [26]. Venous blood was collected by a trained phlebotomist who observed universal precautions in the first-aid area of each school. Participants were seated and resting for collection. Afterwards, they were required to remain in the vicinity for 15 min of observation. Three study investigators who are also practising clinicians (BM, YD, AR) were on-site during the collection of these samples; there were no reportable adverse events. Haemoglobin was analysed on-site using a Sysmex pocH-100i automated Haematology analyser. The other assays were transported and analysed at the Laboratorium Terpadu, Medical Faculty University of Indonesia (ISO17035 accredited). Blood pressure was measured using an automated wrist sphygmomanometer (Omron HEM-6121); this was used to minimise assessment time, enable blood pressure to be measured by non-clinical staff, and to minimise the need to remove clothing for participants. Participants were seated and resting prior to having two measurements taken at one-minute intervals. A third measurement was taken if the systolic reading differed by >10 mmHg or the diastolic by >6 mmHg. To validate this measure, 26 participants were randomly selected from across two schools to have their blood pressure manually measured twice by clinicians BM, YD or AR. Figure 1 reports the Bland-Altmann plots for these two measures of blood pressure, showing that automated measures were largely in limits for systolic and diastolic pressure. There were 611 participants who participated in the school-based study in Jakarta, of whom 455 (75%) consented and participated in the biomarker sub-study.

**Table 4.** Criteria for metabolic syndrome [51]

Metabolic syndrome for this study was defined as central obesity (waist circumference of ≥90 cm for males and ≥80 cm for females), plus two of the following:
Raised triglycerides (≥1.7 mmol/l) Reduced HDL-cholesterol (<1.03 mmol/l in males, <1.29 mmol/l in females) Raised blood pressure (systolic: ≥130 mmHg or diastolic: ≥85 mmHg) Raised HbA1 c (≥5.6%)

#### Formal psychiatric interview

The CESD-R and K10 were formally translated and validated for use in this study [27]. For this task, which included establishing appropriate thresholds, school-based participants from Jakarta were randomly invited to have a formal psychiatric interview (details below). The Mini International Neuropsychiatric Interview for Children and Adolescents (MINI-Kid) was used, with modules including: major depressive episode, dysthymia, panic disorder, separation anxiety disorder, and generalised anxiety disorder [28]. We limited this assessment to school-based adolescents in Jakarta to ensure we could follow-up any clinically concerning findings. We calculated that a sample size of 180 was required to estimate the sensitivity and specificity of a threshold of K10 and CESD-R with a precision of <10%; 196 participated in this module. In each class selected for this module (detailed below), six consenting students were randomly selected (using the RAND command in excel) to participate. To minimise respondent burden, these psychiatric interviews were conducted the day after the main survey. Interviews were administered by child and adolescent psychiatrists (TW, FK) or psychologists in training (supervised by TW and FK) in a private space within the school. Each interview took approximately 30 min.

#### Diet diary

In addition to the nutrition items included in the main survey (Table 3), all school-based participants in Jakarta were invited to download and complete an online 24-hour diet recall diary using the android application ‘KUALA24ʹ (unpublished, co-developed by AR and BW). KUALA24 is specific to Indonesian food and drinks, which allows for a nuanced, culturally appropriate assessment of dietary intake. As only 12 participants logged onto the application and no participant fully completed this assessment, no data were available for analysis.

### Sample size

We estimated a formal required sample size for the school-based sample. Given the complex sampling approach, we estimated that a sample size of approximately 1500 was required to estimate a conservative prevalence of 50% of either NCD outcome or risk, with a margin-of-error of 5% (95% confidence, design effect [deff] = 2.0). Given the unique sampling theory underpinning Respondent-Driven Sampling (RDS), a sample size target of 800 was primarily based on feasibility and budget.

### Sampling strategy and recruitment

In-school adolescents were sampled using multi-stage sampling, while out-of-school adolescents were sampled using RDS.

#### School-based participants

Given the target age range of 16–18 years, we recruited students from grades 10–12 in Indonesia. We randomly selected a total of 24 schools; 12 senior high schools from the 581 public, private and religious senior high schools in Jakarta and 12 from the 987 schools in South Sulawesi (which is a larger geographic province than Jakarta). In consultation with the relevant school principal or administrator, we then identified the number of grades 10, 11 and 12 classes in each school and randomly selected one class from each grade for each school (three classes per participating school). All students of the selected classes were invited to participate if they were eligible: aged 16–18 years, enrolled and attending the school in the previous 90 days; and able to obtain parent/guardian consent. Students were excluded if they had a significant health issue or other reason that might impact on their participation in the study, as decided by the investigator team. At the time of data collection, one school in Jakarta declined to participate due to exam period; given the larger than expected grade sizes and large sample obtained from other schools, this school was not replaced. In total 2,509 school students were invited to participate (consent forms sent home to parents), with 1,337 (53.2%) participating in the school-based survey (611 from Jakarta and 726 from South Sulawesi) (Table 5).

**Table 5.** School-based sample

	Jakarta				South Sulawesi			
	Grade 10	Grade 11	Grade 12	Total	Grade 10	Grade 11	Grade 12	Total
Number of consent forms distributed	414	440	410	**1264**	369	432	444	**1245**
Number of consent forms completed and returned	199	254	175	**628**	218	244	267	**729**
Excluded – did not meet inclusion criteria for age	17	0	0	**17**	2	0	1	**3**
**Completed questionnaire**	**182**	**254**	**175**	**611**	**216**	**244**	**266**	**726**
Consented and eligible for metabolic sub-study	161	200	153	**514**				
Completed metabolic sub-study	157	159	138	**454**				
Consented and eligible for mental health sub-study	149	179	123	**451**				
Randomly selected to complete mental health sub-study	64	84	48	**196**				

This table shows the school-based sample for the questionnaire and sub-studies, across Jakarta and South Sulawesi. Bold values signify total counts.

#### Community-based participants

**Figure 2.**
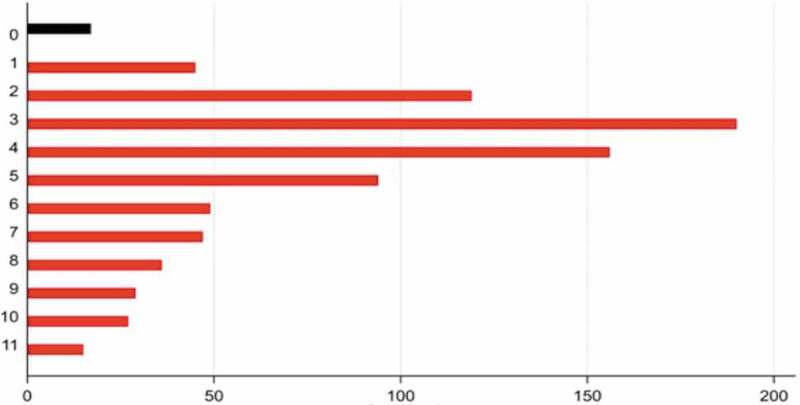
Referral chain depth for respondent-driven sampling (community-based sample)

Respondent-Driven Sampling (RDS) was used to recruit out-of-school adolescents, a chain-based recruitment strategy that is widely used to sample ‘hard to reach’ populations, including adolescents [29,30]. In brief, RDS identified a number of initial ‘seeds’ to participate in the study. These seeds then referred a number of peers to participate (in this study 3 peers were invited), who were each subsequently invited to refer peers to participate. Referrals were managed through the use of coded but de-identified coupons (in our case, with an expiry of 14 days), that linked each participant back to the referrer and the original seed. We recruited seeds across four geographic locations: Central Jakarta, non-central (East) Jakarta, Makassar (urban South Sulawesi) and Jeneponto (remote South Sulawesi). Within each of these four locations, we selected four seeds (16 overall) who represented diversity in terms of gender and the reasons they were not at school (such as homelessness, parenthood, engagement in work or vocational training). These seeds were identified through staff at youth centres, homeless shelters, community health workers, and workplaces. We aimed for 200 responses from each of the four geographic locations (total sample 800). One additional seed was identified in Central Jakarta to achieve this (a total of 17). Eligible participants (aged 16–18 years, not attending school in the 90 days prior, able to obtain parental/guardian consent) completed the same survey as the school-based participants, with additional items relating to the size of their social network. Each participant received a small token for participation and was reimbursed for transportation if required. In total, 824 participants were recruited through the 17 seeds (421 in Jakarta and 403 in South Sulawesi). The most common chain length was three referrals deep; the longest was 11 referrals deep (Figure 2).

This figure shows the distribution of referral chains in respondent-driven sampling in the red bars (black bars are the seeds).

### Procedure

The quantitative component was completed between February and December 2018, with the school-based sample completed first. In Jakarta, school-based assessments begun with registration and confirming parent/guardian consent. Participants then completed the electronic survey within a normal class session (60 min). Over the next 60 minutes, students participated in a ‘lite’ class, with consenting students being called out to have their anthropometry measured and biomarkers collected. In total the assessments took 2 h per class group, making it possible to complete all three grades in a single day visit. To ensure efficiency, we extensively practised the logistics with the team in a ‘mock’ classroom setting. Participants selected and consenting to have a mental health interview were seen on the subsequent day. For the South Sulawesi school-based sample, the inclusion of only the survey and basic anthropometry meant that most could be completed in a single class session. For the community-based sample, community-based hubs were established in the four geographic locations. These were staffed by research assistants with an appointment system.

### Analysis

Quantitative data from the school and community-based samples will be analysed separately owing to their distinct populations and sampling methodologies. Population prevalence (of mental disorder, of metabolic syndrome) and independent exposure effects will be weighted using post-stratification inverse-probability weights and Taylor-linearised variance/standard errors estimation used for inference – accounting for the lack of independence in observations due to the complex sampling methodology. Cross-sectional associations with selected exposures will be estimated using multivariate logistic regression. The RDS estimator will be used to derive and apply the appropriate sampling weights in prevalence and logit regression estimation in the community population and bootstrapped standard errors estimated for inference. Missing data (scale scores and covariates) will be imputed using Multiple Imputation for both prevalence estimation and logit regression analyses in both in- and out-of-school populations.

### Follow-up and referral

Data captured through the psychiatric interview and biomarker assessment were considered of clinical relevance. Following psychiatric interviews, arrangements were made for participants who were assessed to have significant psychiatric symptoms to be followed up through the Department of Psychiatry, Universitas Indonesia and/or their local doctor. For the biomarker assessments, any participant with results that met criteria for the metabolic syndrome (as defined in Table 4), hypertension alone (Table 4), impaired glucose tolerance (≥5.7%), vitamin D deficiency (<15 ng/ml) or anaemia (Haemoglobin <130 g/L males, <120 g/L females, <100 g/L pregnant) was followed up by a letter sent to their parent/guardian with information about the result that contained advice to consult with their local doctor.

No follow-up or referral was offered to any individual on the basis of responses made in the self-reported survey. Instead, at the end of the survey, all participants were provided with written information about local health and mental health services. Participants were also encouraged to speak to any of the study staff if they were distressed.

## Ethical approval

Ethical approval was provided by the Alfred Hospital Health Research Ethics Committee in Australia (approval 114/17) and by Ethics Committee of the Faculty of Medicine, Universitas Indonesia (approval 714/UN2.F1/ETIK/2017). Three issues were discussed at length with the Australian ethics committee. The first is related to consent for participation. Despite recognition of the emerging capacities of adolescents older than 16 years [23] and potential impact on recruitment, a condition of ethical approval was that parental consent needed to be obtained for all participants. In the case of homeless young people, it was considered acceptable to obtain this consent from the manager of the homeless shelter. Secondly, there was concern that items relating to substance use may incriminate or socially marginalise young people who reported use. Our response to the ethics committee (and participants) was to assure them that the self-report surveys were completely anonymous and did not collect any identifying information (including date of birth). To enable follow-up of biomarker results, contact details were obtained and stored separately and destroyed once follow-up was complete. The third issue discussed was around concerns about the need to follow-up reports of self-harm. However, as the items enquired about self-harm over the preceding year (not current ideation), it was agreed that we were not measuring acute risk. As outlined above, all respondents received information about local mental health services.

## Planned analyses

We plan to analyse the qualitative data to report the concepts, perceived determinants and responses to poor mental health and overweight, respectively, from the perspective of Indonesian young people. This analysis and new understandings will then frame the analysis of quantitative data where we aim to report the population prevalence, correlates and inter-relationship of poor mental health and metabolic syndrome.

## Discussion

This study will substantially advance the assessment of NCD risk and outcomes amongst in-school adolescents as well as more vulnerable adolescents. It demonstrates that quality, objective measurement is acceptable and feasible, including the collection of biomarkers in a school-based setting. Furthermore, we have shown that comparable data can be collected from in-school and out-of-school adolescents which allows a more comprehensive measure of NCD burden and risk. This study particularly demonstrates the value of formative qualitative enquiry that privileges the voice and perspectives of young people themselves. This helped to ensure we aligned the subsequent quantitative measures with the perceived mental health concerns of Indonesian adolescents, and will also, in due course, inform considerations around the appropriateness and acceptability of responses.

This study addresses substantial data gaps around metabolic risk in Indonesia, which will help to identify opportunities for intervention early in the life course where interventions are likely to be most effective [16]. Available data on the metabolic syndrome in Indonesia are predominantly from adult samples [31]. For example, adolescents were absent from Indonesia’s most recent WHO STEPS surveillance, which was conducted in 2006 and only sampled those over the age of 25 years [32]. Indonesia’s ongoing NCD surveillance program Pos Pembinaan Terpadu (POSBINDU) is largely drawn from health services, likely to under-sample adolescents who are asymptomatic.

This study also makes important contributions around mental health, an area where data are desperately lacking, not only in Indonesia [9]. While both the Global School Health Survey and RISKESDAS include measures of mental health in Indonesian adolescents, neither survey includes any measure that has been formally validated for clinically significant mental health problems [33,34]. Furthermore, the sampling frames of these surveys (school and home) are highly likely to exclude those at greatest risk. Indeed, the specific focus on vulnerable adolescents in this study (a pervasively neglected group) is a major strength.

Previous studies in Viet Nam and China have measured the metabolic syndrome amongst adolescents at school [35,36]. Similarly, studies in Viet Nam and Thailand have validated mental health scales and sampled adolescents from both school or community settings [37,38]. What is unique to the current study is the robustness and concurrent measurement of mental health and metabolic risk. A particular strength of this approach is that it will enable better understanding of any relationship between these two NCDs, as well as other important exposures such as victimisation, connectedness and body image as measured through scales included in this study. In addition to insights about pathogenesis, such knowledge could also help frame appropriate public health responses. The sampling of a large number of vulnerable out-of-school adolescents is expected to be of major interest. Understanding the differential prevalence of mental health and metabolic risks is expected to inform population estimates, as well as the need for actions in different settings.

Beyond the quality of measurement and sampling, a strength of this study is that from its inception, it has been a collaborative effort that resulted in shared learnings and capacity development between researchers in both countries. The importance of face-to-face meetings cannot be underestimated as a critical foundation of this collaboration. However, regular (and often informal) communication through closed social media (WhatsApp) has been an equally important aspect of this study that has helped foster meaningful, trusting relationships.

The study has important limitations. Qualitative enquiry used focus group discussions (FGDs) as we hoped to capture a broad range of perspectives; however, stigma related to mental health (and obesity) may have limited discussion. To mitigate this risk, we emphasised that participants should talk about the issues broadly (not their individual experiences), commencing each FGD with an ‘ice-breaker’ to build rapport. The measurement of metabolic syndrome was limited by the non-fasting nature of serum samples, which was required for pragmatic reasons. Of the different measures, blood glucose is most sensitive to fasting status, which has been largely mitigated by the use of HbA1c. We also used an automated wrist sphygmomanometer to measure blood pressure. Reassuringly, our validation exercise found that wrist measurement provided reasonably consistent blood pressure recordings when compared to physician measurements. While we would have preferred to have included a wider sample of adolescents for psychiatric interview, we felt that the priority had to be that of safety, which led to our more limited focus on in-school adolescents from Jakarta. Engagement with the online diet recall survey was also limited, likely reflecting the need for young people to complete this in their own time, availability on the android platform only, and potential concern around data usage. We had anticipated that participation in this online module may be biased and included key nutritional measures in the core survey as a safeguard.

In conclusion, the data generated from this study will strengthen our understanding of NCDs amongst adolescents in Indonesia, the world’s third most populous country that is central to the prosperity of the Asia Pacific region. We hope that the methods and measures developed here may help strengthen future NCD surveillance systems in Indonesia, and that the study may serve as an example of how to strengthen NCD measurement for adolescents in low- and middle-income countries more generally.

## Paper context

Data on NCDs amongst adolescents are limited in quality, especially in low- and middle-income countries, posing a barrier to effective investment and action. We designed a research study to objectively measure mental disorder (a key outcome) and metabolic syndrome (a key risk) amongst adolescents in Indonesia, sampling both in-school and community-based adolescents. This paper demonstrates that high quality, objective measure of NCD in adolescents is acceptable and feasible, including in settings like Indonesia.

## References

[1] Global Burden of Disease DALYs and Hale Collaborators. Global, regional, and national disability-adjusted life-years (DALYs) for 359 diseases and injuries and healthy life expectancy (HALE) for 195 countries and territories, 1990–2017: a systematic analysis for the global burden of disease study 2017. Lancet. 2018;392(10159):1859–14. Epub 2018/11/13. PubMed PMID: 30415748; PubMed Central PMCID: PMCPMC6252083. 30415748 10.1016/S0140-6736(18)32335-3 PMC6252083

[2] United Nations. Time to deliver: third UN high-level meeting on non-communicable diseases. 2018.

[3] Patton GC, Sawyer SM, Santelli JS, et al. Our future: a Lancet commission on adolescent health and wellbeing. Lancet. 2016;387(10036):2423–2478. Epub 2016/05/09. PubMed PMID: 27174304; PubMed Central PMCID: PMC5832967. 27174304 10.1016/S0140-6736(16)00579-1 PMC5832967

[4] WHO. The world health report 2002: reducing risks, promoting health life. Geneva: World Health Organization; 2002.

[5] Azzopardi PS, Hearps SJC, Francis KL, et al. Progress in adolescent health and wellbeing: tracking 12 headline indicators for 195 countries and territories, 1990–2016. Lancet. 2019;393(10176):1101–1118. PubMed PMID: S0140673618324279 30876706 10.1016/S0140-6736(18)32427-9 PMC6429986

[6] Nugent R, Bertram MY, Jan S, et al. Investing in non-communicable disease prevention and management to advance the sustainable development goals. Lancet. 2018;391(10134):2029–2035. PubMed PMID: S0140673618306676 29627167 10.1016/S0140-6736(18)30667-6

[7] Patton GC, Olsson CA, Skirbekk V, et al. Adolescence and the next generation. Nature. 2018;554(7693):458–466. Epub 2018/02/23. PubMed PMID: 29469095 29469095 10.1038/nature25759

[8] Li Z, Li M, Patton GC, et al. Global development assistance for adolescent health from 2003 to 2015. JAMA Network Open. 2018;1(4):e181072. Epub 2019/ 01/16. PubMed PMID: 30646101; PubMed Central PMCID: PMCPMC6324521. 30646101 10.1001/jamanetworkopen.2018.1072 PMC6324521

[9] Erskine H, Baxter A, Patton G, et al. The global coverage of prevalence data for mental disorders in children and adolescents. Epidemiol Psychiatr Sci. 2017;26(4):395–402. 26786507 10.1017/S2045796015001158 PMC6998634

[10] Howie SR. Blood sample volumes in child health research: review of safe limits. Bull World Health Organ. 2011;89(1):46–53. Epub 2011/ 02/25. PubMed PMID: 21346890; PubMed Central PMCID: PMCPMC3040020. 21346890 10.2471/BLT.10.080010 PMC3040020

[11] Azzopardi P, Kennedy E, Patton G. Data and indicators to measure adolescent health, social development and well-being. Florence: UNICEF Office of Research – Innocenti; 2017. (Innocenti Research Briefs 2017-04; Methods: Conducting research with adolescents in low- and middle-income countries, no.2).

[12] Directorate General Of Disease Control And Environmental Sanitation. National strategic action plan for the prevention and control of noncommunicable diseasess (RAN PP-PTM) 2016–2019. Indonesia: Ministry Of Health Of The Republic Of Indonesia; 2016.

[13] Mboi N, Surbakti M, Trihandini I, et al. On the road to universal health care in Indonesia, 1990–2016: a systematic analysis for the global burden of disease study 2016. Lancet. 2018;392(10147):581–591. Epub 2018/ 07/03. PubMed PMID: 29961639; PubMed Central PMCID: PMCPMC6099123 29961639 10.1016/S0140-6736(18)30595-6 PMC6099123

[14] Blum RW. Distressed communities as a breeding ground for noncommunicable conditions. J Adolesc Health. 2014;55(6 Suppl):S4–5. Epub 2014/12/03. PubMed PMID: 25454002. 25454002 10.1016/j.jadohealth.2014.09.009

[15] Mokdad AH, Forouzanfar MH, Daoud F, et al. Global burden of diseases, injuries, and risk factors for young people’s health during 1990–2013: a systematic analysis for the global burden of disease study 2013. Lancet. 2016;387(10036):2383–2401. PubMed PMID: 27174305 27174305 10.1016/S0140-6736(16)00648-6

[16] Cini K, Ancha A, Sawyer S, et al. Towards a comprehensive NCD reporting framework for Indonesia. Melbourne (Australia): Australia-Indonesia Centre, Health Cluster; 2018.

[17] Pervanidou P, Chrousos GP. Stress and obesity/metabolic syndrome in childhood and adolescence. Int J Pediatr Obes. 2011;6(Suppl 1):21–28. Epub 2011/09/16. PubMed PMID: 21905812. 10.3109/17477166.2011.615996 21905812

[18] Zimmet P, Alberti K, Stern N, et al. The circadian syndrome: is the metabolic syndrome and much more! J Intern Med. 2019. Epub 2019/ 05/14. DOI:10.1111/joim.12924. PubMed PMID: 31081577. PMC6851668 31081577

[19] World Health Organization ROfS-EA. Global Youth Tobacco Survey (GYTS): Indonesia report, 2014. New Delhi: WHO-SEARO; 2015.

[20] Cheng Y, Li X, Lou C, et al. The association between social support and mental health among vulnerable adolescents in five cities: findings from the study of the well-being of adolescents in vulnerable environments. J Adolesc Health. 2014;55(6 Suppl):S31–8. Epub 2014/ 12/03. PubMed PMID: 25454000 25454000 10.1016/j.jadohealth.2014.08.020

[21] Badan Pusat Statistik Kabupaten Gowa. Kabupaten Gowa Dalam Angka. BPS-Statistics of Gowa Regency. 2018.

[22] Badan Pusat Statistik Kabupaten DKI Jakarta. Kabupaten DKI Jakarta Dalam Angka. BPS-Statistics of DKI Jakarta. 2018.

[23] Santelli J, Haerizadeh S, MCGowan T. Inclusion with protection: obtaining informed consent when conducting research with adolescents. Florence (Italy): UNICEF Innocenti; 2017.

[24] Van Dam NT, Earleywine M. Validation of the Center for Epidemiologic Studies Depression Scale–Revised (CESD-R): pragmatic depression assessment in the general population. Psychiatry Res. 2011;186(1):128–132. Epub 2010/ 09/17. PubMed PMID: 20843557. 20843557 10.1016/j.psychres.2010.08.018

[25] Kessler RC, Barker PR, Colpe LJ, et al. Screening for serious mental illness in the general population. Arch Gen Psychiatry. 2003;60(2):184–189. Epub 2003/02/13.PubMed PMID: 12578436 12578436 10.1001/archpsyc.60.2.184

[26] World Health Organization. Use of glycated haemoglobin (HbA1c) in the diagnosis of diabetes mellitus: abbreviated report of a WHO consultation. Geneva: World Health Organization; 2011. Available from: https://www.ncbi.nlm.nih.gov/books/NBK304267/26158184

[27] Tran TD, Kaligis F, Wiguna T, et al. Screening for depressive and anxiety disorders among adolescents in Indonesia: formal validation of the centre for epidemiologic studies depression scale – revised and the Kessler psychological distress scale. J Affect Disord. 2019;246:189–194. PubMed PMID: S016503271831752X 30583144 10.1016/j.jad.2018.12.042

[28] Sheehan DV, Sheehan KH, Shytle RD, et al. Reliability and validity of the Mini International Neuropsychiatric Interview for children and adolescents (MINI-KID). J Clin Psychiatry. 2010;71(3):313–326. Epub 2010/ 03/25. PubMed PMID: 20331933 20331933 10.4088/JCP.09m05305whi

[29] Gile KJ, Handcock MS. Respondent-driven sampling: an assessment of current methodology. Sociological Methodol. 2010;40(1):285–327. PubMed PMID: 22969167. 10.1111/j.1467-9531.2010.01223.x PMC3437336 22969167

[30] Decker MR, Marshall B, Emerson M, et al. Respondent-driven sampling for an adolescent health study in vulnerable urban settings: a multi-country study. J adolesc health. 2014;55(60):S6–S12. PubMed PMID: PMC4443701. 25454005 10.1016/j.jadohealth.2014.07.021 PMC4443701

[31] Schröders J, Wall S, Hakimi M, et al. How is Indonesia coping with its epidemic of chronic noncommunicable diseases? A systematic review with meta-analysis. PLoS One. 2017;12(6):e0179186–e. PubMed PMID: 28632767 28632767 10.1371/journal.pone.0179186 PMC5478110

[32] Centre for Disease Control Research and Development NMoH, Republic of Indonesia, World Health Organization. Monitoring and evaluation of the integrated community-based intervention for the prevention of NCD in Depok. West Java (Indonesia); 2006.

[33] Indonesian Ministry of Health. Basic health research (RISKESDAS). Jakarta: Ministry of Health; 2013.

[34] WHO and Centers for Disease Control and Prevention. Global school health survey 2012. Available from: https://www.who.int/ncds/surveillance/gshs/en/

[35] Nguyen TH, Tang HK, Kelly P, et al. Association between physical activity and metabolic syndrome: a cross sectional survey in adolescents in Ho Chi Minh City, Vietnam. BMC Public Health. 2010;10:141. Epub 2010/ 03/20. PubMed PMID: 20236509; PubMed Central PMCID: PMCPMC2847981. 20236509 10.1186/1471-2458-10-141 PMC2847981

[36] Xu YQ, Ji CY. Prevalence of the metabolic syndrome in secondary school adolescents in Beijing, China. Acta Paediatr. 2008;97(3):348–353. Epub 2008/02/27. PubMed PMID: 18298784. 18298784 10.1111/j.1651-2227.2008.00665.x

[37] Nguyen DT, Dedding C, Pham TT, et al. Depression, anxiety, and suicidal ideation among Vietnamese secondary school students and proposed solutions: a cross-sectional study. BMC Public Health. 2013;13:1195. Epub 2013/ 12/18. PubMed PMID: 24341792; PubMed Central PMCID: PMCPMC3878548. 24341792 10.1186/1471-2458-13-1195 PMC3878548

[38] Akiyama T, Win T, Maung C, et al. Mental health status among Burmese adolescent students living in boarding houses in Thailand: a cross-sectional study. BMC Public Health. 2013;13:337. Epub 2013/ 04/17. PubMed PMID: 23587014; PubMed Central PMCID: PMCPMC3636114 23587014 10.1186/1471-2458-13-337 PMC3636114

[39] Centers for Disease Control and Prevention. Youth Risk Behaviour Surveillance System (YRBSS) 2018. Available from: https://www.cdc.gov/healthyyouth/data/yrbs/questionnaires.htm

[40] Ravens-Sieberer U, Gosch A, Rajmil L, et al. The KIDSCREEN-52 quality of life measure for children and adolescents: psychometric results from a cross-cultural survey in 13 European countries. Value Health. 2008;11(4):645–658. Epub 2008/01/09. PubMed PMID: 18179669 18179669 10.1111/j.1524-4733.2007.00291.x

[41] Varni JW, Seid M, Rode CA. The PedsQL: measurement model for the pediatric quality of life inventory. Med Care. 1999;37(2):126–139. Epub 1999/ 02/19.PubMed PMID: 10024117. 10024117 10.1097/00005650-199902000-00003

[42] Mutale GJ, Dunn AK, Stiller J, et al. Development of a body dissatisfaction scale assessment tool. New Sch Psychol Bull. 2016;13(2):47–57.

[43] Roberts C, Freeman J, Samdal O, et al. The Health Behaviour in School-aged Children (HBSC) study: methodological developments and current tensions. Int J Public Health. 2009;54(2):140–150. 19639259 10.1007/s00038-009-5405-9 PMC2732766

[44] Currie C, Nic Gabhainn S, Godeau E. The international HBSC network coordinating committee. The health behaviour in school-aged children: WHO collaborative cross-national (HBSC) study: origins, concept, history and development 1982–2008. Int J Public Health. 2009;54(2):131–139. 10.1007/s00038-009-5404-x 19639260

[45] WHO and Centers for Disease Control and Prevention. Global Youth Tobacco Survey (GYTS) 1998. Available from: https://www.who.int/tobacco/surveillance/gyts/en/

[46] Finkelhor D, Hamby SL, Ormrod R, et al. The Juvenile victimization questionnaire: reliability, validity, and national norms. Child Abuse Negl. 2005;29(4):383–412. Epub 2005/ 05/27. PubMed PMID: 15917079. 15917079 10.1016/j.chiabu.2004.11.001

[47] Schwarzer R, Jerusalem M. Generalised self-efficacy scale. In: Weinman J, Wright S, Johnston M, editors. Measures in health psychology: a user’s portfolio causal and control beliefs. Windsor (UK): NFER-NELSON; 1995. p. 35–37.

[48] Lee R, Robbins S. Measuring belongingness: the social connectedness and the social assurance scales. J Couns Psychol. 1995;42(2):232–241.

[49] Arthur MW, Hawkins JD, Pollard JA, et al. Measuring risk and protective factors for substance use, delinquency, and other adolescent problem behaviors. The communities that care youth survey. Eval Rev. 2002;26(6):575–601. Epub 2002/12/06. PubMed PMID: 12465571. 12465571 10.1177/0193841X0202600601

[50] Mujahid MS, Diez Roux AV, Morenoff JD, et al. Assessing the measurement properties of neighborhood scales: from psychometrics to ecometrics. Am J Epidemiol. 2007;165(8):858–867. Epub 2007/ 03/03. PubMed PMID: 17329713. 17329713 10.1093/aje/kwm040

[51] International Diabetes Federation. The IDF consensus worldwide definition of the metabolic syndrome. Brussels (Belgium): IDF; 2006.

